# Experiencing Rhythm in Dance

**DOI:** 10.3389/fpsyg.2022.866805

**Published:** 2022-05-30

**Authors:** John M. Wilson, Matthew Henley

**Affiliations:** ^1^School of Dance, University of Arizona, Tucson, AZ, United States; ^2^Dance Education Program, Department of Arts and Humanities, Teachers College, Columbia University, New York, NY, United States

**Keywords:** dance, rhythm, experience, energy, H’Doubler

## Abstract

In this article, two dance educators offer a definition of rhythm from both educational and performance perspectives and discuss pedagogical practices that waken students’ awareness to rhythm as a lived-experience over which they have creative control. For the dancer, in the midst of the dance, rhythms are, in the words of Margaret H’Doubler, recurring patterns of measured energy. These patterns are nested in scales from the moment-to-moment shifts in muscular contraction and release to the rise and fall of dramatic tension in a performed dance. This approach to rhythm runs counter to many dance students’ studio-based training in which rhythm is equated to synchronizing accents to a specific meter. The authors describe pedagogical practices in the studio that foster engagement with rhythm as lived-experience. Drawing attention to their kinesthetic experience while moving, students are encouraged to modulate levels of exertion embedded in the qualities of movement they are experiencing. As varying levels of exertion are attended to across temporal durations, students notice patterns as they emerge and recur. This attention to recurring patterns of measured exertion is, the authors claim, the lived-experience of rhythm in dance.

## Introduction

Rhythm is a familiar enough word; yet its meaning depends on the user or, more precisely, on how the user uses it. Rhythm means one thing to a farmer and another to a newspaper editor. Nonetheless, each user understands, in a general way, what the other user is referring to when they use the word. Rhythm is not a thing. Nor does it refer to a thing. Rhythm is a phenomenon like time and space. It extends well beyond the particulars of one’s work. That makes it difficult to define. A popular answer to the question, “What is rhythm?” could be, “I can’t define it, but I know it when I see, hear, or feel it.” In short, rhythm is experiential, palpable to the senses though elusive to the analytical mind.

In the psychological literature, rhythm has primarily been considered in the domains of music and speech perception where it is generally defined as patterns of relative durations between notes, tones, or other acoustic events (Levitin et al., 2018). Cummins (2009) argues that these definitions inadequately account for the ecological role of rhythm. Whether in music or speech perception, rhythms are not mere acoustic waves available for passive perception, rather they serve as affordances for the coordination of stimulus and action. This coordination is referred to as entrainment, in which rhythm acts a coordinating force between dynamic systems.

Though entrainment between action and auditory stimuli serves an important role for ecological purposes of survival and social coordination, rhythm can also be perceived by sensory modalities other than audition including haptic, proprioceptive, visual, and vestibular systems (Levitin et al., 2018). Phillips-Silver et al. (2010) describe several forms of entrainment, one of which is self-entrainment, in which the stimulus to which an agent responds is their own rhythmic output. Rhythm is perceived not as a series of acoustic or visual events, but as the “energetic turning points in the [agent’s] motion” (Waterhouse et al., 2014). Explicit awareness of the sense of self-movement, or kinesthesis, as a mode of self-coordination is central to the practice of dancing. Rhythmic awareness and practice sit at the center of many dances, wherein there is a mutual coordination between musician and dancer (Cruz Banks, 2021). In the historical traditions of modern and post-modern concert dance, which are the authors’ areas of expertise, educators and choreographers sought to separate music as a source of the dancer’s energetic coordination and prioritize the self as the source of rhythmic output. Thought of in this way, rhythmic awareness is a form of self-entrainment, in which the dancer attends to energetic turning points created by the dancing in which they are engaged.

In this article we describe how three generations of dance educators sought to understand and develop this approach to rhythm. The article begins with a brief history of Margaret H’Doubler’s development of the definition: “rhythm is measured energy” (H’Doubler, 1921, 1932). We then describe how a student of H’Doubler’s, and co-author of this article John Wilson, extended H’Doubler’s definition through empirical research on primary and transitional movement qualities. In the final section, a student of Wilson’s, and co-author of this article Henley, describes how these definitions have been informed, clarified, and extended in his own teaching practice by phenomenological and process philosophy. In Supplementary Appendix, we offer practical suggestions for readers to begin to develop their own awareness of rhythm as energy measured through the sense of self-movement.

## The Search for Meaning in Dance Education

H’Doubler was neither a dancer nor a choreographer but she is celebrated in the dance world as the “pioneer” who created the first dance degree program in America at the University of Wisconsin in Madison. The year was 1926. This feat was followed by the creation of a master’s degree program in 1928 leading to an M.S. or M.A. depending on the curriculum “track” the student pursued (Ross, 2000). H’Doubler (the H is silent) was the founder of an intellectual tradition that defines the facts and principles of the experience of dancing. Rhythm is but one of those principles (H’Doubler, 1940).

In 1916, H’Doubler,^1^ an instructor in the Department of Women’s Physical Education at University of Wisconsin, Madison was tapped by her director, Blanche Trilling, to take a leave of absence and go to New York City to “find out what this new dancing is all about. See if it has value for our young women” (Wilson et al., 2006). She departed for New York and Columbia University’s Teachers College.^2^ Teachers College was home to new ideas in experimental education that were gaining currency in the country. One of the experiments was a dance program designed and led by an adjunct faculty member, Gertrude K. Colby (1874–1960) and her associate, Bird Larson^3^.

H’Doubler would have been aware of Colby’s efforts but, for some reason, she recorded and spoke very little about that. Members of “Miss HuhDee’s” (an affectionate appellation) graduate student seminars at UW-M after she retired in 1954 assumed that she felt Colby relied too much on familiar folk-dance rhythms for her students’ “free expression.”^4^ H’Doubler had not yet figured out an alternative to teaching dance by “steps in time.” Nonetheless, Colby’s speculative ideas about the significance of rhythm in human movement very much coincided with H’Doubler’s developing ruminations on the subject. As her experimentation developed over the years, Colby often expressed her belief that dance education should spring from each child’s “natural” body rhythms—that each child is endowed at birth with her or his unique, personal rhythms and that those rhythms “should be the foundation of her [Colby’s] educational program.” Her guiding principle was: “By making ourselves free instruments of expression, rhythmically unified, we are enabled to express in bodily movement the ideas and emotions which come from within. ‘We dance ideas, not steps”’ (Colby, 1922, pp. 7–8)^5^.

The identity of Colby’s thoughts within H’Doubler’s cannot be missed. Those thoughts become pronouncements in the latter’s classic book, *Dance: A Creative Art Experience* (1940). The heading for Chapter 2, “The Province of Dance,” states: “Art as creative expression has its source within man’s physical, mental, and emotional structure; dance therefore is the heritage of all mankind.” Subsequent chapters 3–7 lay out how this can be realized in education. And, by implication, that was not only in dance education but general education.^6^

Trilling also suggested H’Doubler attend a class taught by Alys Bentley (1869–1951), a children’s music teacher in New York City who was regarded as an innovator in her teaching methods. First, the children were instructed to lie on the floor as music played. The children were to react to what they felt, emotionally, by moving their bodies. H’Doubler joined in this exercise and suddenly realized the key to what would be basic to her dance classes:

“(…) she got us down on the floor and had us do some rotations and work with some flexions and extensions (…) but not talking to us or telling us why or anything about it. But then it came just like a flash: ‘Of course! Get on the floor where we’re away from the pull of gravity then work out what really are the structural changes of position of the body when it can move freely!’ And then I commenced to get really excited.”^7^

## The Progressive Dance Class

The necessary parts of H’Doubler’s thinking at last coalesced. From childhood her temperament was that of a scientist. Her father, whom she followed like his shadow, was an inventor with a dozen or more patents in his name. Her academic major was biology. She understood human movement as a kinesiologist does. She also understood motor perception and kinesthesis and its linkage with emotions. From her required undergraduate P.E. classes she knew how to perform folk dance and eurhythmics (Findlay, 1971). She was committed to progressive education, and she preferred teaching in the Socratic method with interactive dialogue.

A full sized, manufactured human skeleton hung on a rolling pole in every class she taught in her long career. To begin the physical activity of a class, following a short anatomical lecture for the day, the recumbent students spontaneously explored joint movements with their eyes closed. Continuing the improvisation she instructed them to give their attention to the kinesthetic feedback they experienced from muscle contraction and release. The classes ended with short, student-taught dance pieces followed by class discussion on the day’s learning experiences.

Transferring to standing posture, the students were instructed to continue the joint and muscle explorations that had begun on the floor. Shortly after that part of the activity the students, individually, created their own rhythms to support the movements they had experienced kinesthetically and emotionally. Eventually H’Doubler had each student demonstrate what she or he had created. After a brief response from the witnessing class, the student’s creation was repeated. The accompanist joined in spontaneously with piano, percussion, or wordless song (Ross, 2000).

All elements of a progressive “new dance” experience were in logical sequence for teaching and artmaking. H’Doubler’s definition for rhythm served her classes, but posed an important question: how is energy measured? In the next section, we describe how Wilson addressed that question.

## The Movement Qualities

H’Doubler retired from the Department of Physical Education at the University of Wisconsin-Madison as Professor Emerita in 1954. She was often invited by the department faculty to return to the campus to teach classes and lead open discussions. Seminars for graduate students were conducted in the studio where they experienced her unique movement classes, followed by in-depth discussions on topics that the students brought up. Questions about rhythm and the teaching thereof were frequent. H’Doubler’s succinct definition, “measured energy,” was particularly provocative, both on the practical and the theoretical level. How can energy *be* measured? Energy within the body is constantly in flux so is not *exertion* what we are concerned with, not energy? We are concerned with *how* exertion is used—how it is manifested in our movements. This question deserved exploration. It deserved empirical evidence. With H’Doubler’s encouragement, a team of graduate classmates set out to find that evidence.

The first subset of questions was to find what the phrase *movement qualities* referred to. Every dance teacher and choreographer uses the term “quality” and its plural form. But there is no set or universal vocabulary to describe all the qualities. The most often named as qualities are “swinging,” “percussive,” “sharp,” “soft,” “smooth,” “heavy,” “light.” These and dozens more can be useful—even essential—instructions to dancers depending on the circumstances. Rudolf von Laban (1879–1958), Hungarian choreographer and dance theorist, developed a vocabulary for movement *efforts*. Many books by Laban and his followers are in publication or rare books collections. Most of these are organized as manuals for teaching and several are theoretical (Laban and Lawrence, 1947; Newlove and Dalby, 2004).

In his system for teaching Laban uses the term “effort” rather than energy. But his “Eight Efforts” refer to biomechanical actions rather than to root neurological qualities. The Eight Efforts are: punch, slash, dab, flick, press, wring, glide, and float. These “efforts” imply but do not specifically refer to “raw” exertion. A member of the research team added the word raw to exertion to differentiate what they were searching for from efforts by which Laban identified actions, a critical distinction in the pursuit of movement qualities.

A second question arose: are there a limited number of basic movement qualities on which all variations are based? How those were to be found was the challenge. The team began by using very thin electrodes inserted in specific muscles, such as rectus femoris that extends the knee and its antagonist, biceps femoris, that assists in flexing the knee. The electrodes were to record electrical charges from the paired muscles on oscillograph paper as one, then the other, was contracted and the knee articulated.

The hope was that a distinctive “profile” of the charges would appear on the paper as the paired muscles contracted and released in different degrees in different trials. Would the amplitudes of the two contractions appear synchronously or asynchronously? Would frequencies coincide, overlap, or not coincide? The answer was null. The markings on the paper were almost black. No profiles appeared. In 1966, when the test trials were performed, electronic equipment was primitive; also, the electrode-to-oscillograph means to determine the profiles of movement qualities was, frankly, ridiculous. Not to be discouraged, however, the team conceived of another approach to answer the question: direct observation—empiricism, pure and simple.

Attempts were made to record moving dancers on film. Videotaping had yet to be invented. A few film strips were successfully made that revealed interesting images. However, the process was expensive and slow. The project was revised again, this time to direct observation of improvised movement with subsequent discussions among select panel members on qualities and how they defined them. The dancers, too, joined the discussions. Four of these sessions were staged, each session with a new set of five dancers at three levels of expertise, beginning to advanced.

At each session the observers—always the same four, including the advanced technique teacher, Louise Kloepper—came close to consensus on what recurring, primary movement qualities they saw and what to name them. A swinging quality was the most frequently performed by the dancers. Second in frequency was a smooth, gradual quality. Third was a percussive quality. One of the instructions to the dancers from time to time was to increase the degree of exertion they “put into” their movements. Almost always, that created moments of percussion; but the more advanced dancers began to experiment with the slow, smooth movement quality increasing to a shaking or vibratory quality as they increased exertion. This was a major discovery for the observers and the dancers alike. Greater or lesser exertion seemed to be the key to identifying different qualities.

After the four sessions were done the team and observers decided to have another two sessions with only advanced dancers participating. Coached now and again to speed up or slow down, or to increase or decrease exertion and yet maintain the quality they were working in at the time, the dancers had many discoveries that the observers witnessed as well. It was a “eureka!” moment. Speed and degree of exertion appeared to be independent factors in the performance of movement qualities. Smooth, for example, could be performed quickly, depending on the skill of the performer. Smooth did not have to be slow to be smooth. Swinging could be performed relatively slowly with high or low exertion. Percussive movement could be performed with a low level of exertion, etc.

These discoveries greatly increased the aesthetic value of the performances according to the observers and in the emotional responses of the dancers. This discovery—that speed of execution and degree of exertion—are independent factors in the performance of movement qualities was remarkable. It supported H’Doubler’s thesis that dance is a creative art experience. A dancer not only can perform a form but interpret it. The heightened kinesthetic experience of the dancers and the heightened aesthetic experience of the observers gave powerful credence to her proclamation. Moreover, it concurred with one of her favorite mottos: “Science cannot make art, but it can contribute to a more truthful art.”

The observing panel came to two conclusions. First, there are four primary movement qualities: sustained, pendulous, abrupt, and vibratory. These four are basic to all qualities of movement and are essential to ordinary living. This was obvious for sustained, pendulous, and abrupt; but vibratory seemed outside normal living experiences—until someone stated, “but when I’m cold or very angry I vibrate; and what about Katharine Hepburn’s chin when she is getting ready to cry?” Vibratory quality did indeed belong in the four basic movement qualities.

The second, conclusive observation by the panel was that all four of these neurologically basic qualities could be *maintained* for several seconds or longer, depending on the energy (yes, *energy* in this usage) available to the performer, without slipping into a different quality. And yet, according to the dancers, there seemed to be (felt to be) other qualities that “tied together” the basic ones. A favorite, kinesthetically and emotionally, was a transition between sustained and pendulous, going either way. They called it “suspended.” The difference between these qualities and the primary ones was that they could not be maintained; they were self-extinguishing—transitional.

**FIGURE 1:**
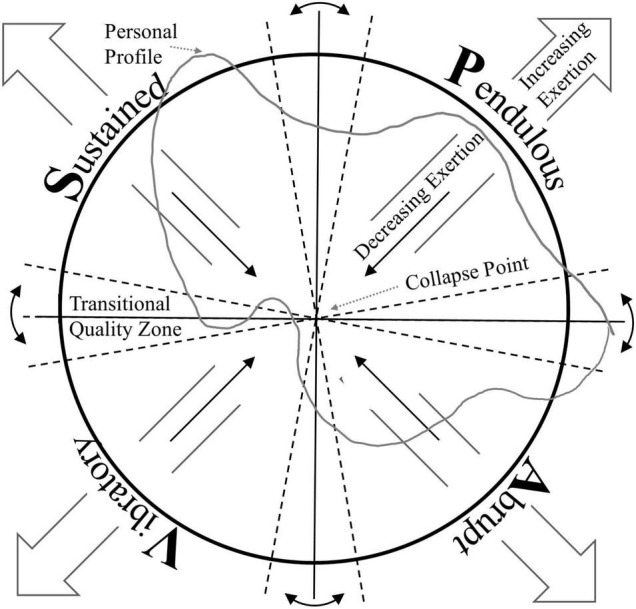
The Autosphere: a graphic depiction of the primary qualities (pendulous, sustained, abrupt, and vibratory) and their transitional zones (e.g., sustained-pendulous). Perceived exertion is indicated by distance from the center or collapse point. Personal profile is an example of a hypothetical student’s reflection on their own movement style.

The consensus of the panel and the dancers was that there are four primary (basic) qualities. Transitional qualities occur in the six zones between the four primary qualities: sustained-pendulous, pendulous-abrupt, abrupt-vibratory, vibratory-sustained, sustained-abrupt, and pendulous-vibratory (see Figure 1, the Autosphere and Supplementary Appendix for suggestions on use of the Autosphere). The characteristics of the primary qualities were also agreed upon:

a. Sustained: continuous movement with no acceleration or deceleration.
b. Pendulous: oscillating movement continuously accelerating and decelerating.
c. Abrupt: movement continuously starting and stopping.
d. Vibratory: continuous rapid quaking as antagonistic muscles contend against each other.

## The Measure of Movement Qualities Through Time is Rhythm

The Autosphere (Figure 1) is an aid to exploring and experiencing the full range of the movement qualities. The two-dimensional circular image represents a sphere in which the primary and transitional movement qualities are depicted in a theoretical relationship. Each of the four primary (i.e., basic) qualities occupies one quarter of the two-dimensional image. At the center of the circle is a non-dimensional point that represents the collapse of all body energy. The qualities extend outward from that “dead center.” The two-legged arrows indicate increasing exertion, even to and beyond the circle depending on the exertion of which the performer is capable. The single legged arrow indicates diminishing exertion toward the collapse point.

At the borders of conjunction between the primary qualities are zones in which the transitional qualities occur. Between sustained and pendulous, for example, is the zone of suspension, going either way from sustained to pendulous or from pendulous to sustained. Between abrupt and vibratory is the zone commonly called percussive; from vibratory to abrupt is typically called explosive, from abrupt to vibratory implosive. Vibratory to sustained is commonly referred to as a releasing quality, from sustained to vibratory a containing quality. Note that in a sphere pendulous and vibratory have a common border as do sustained and abrupt, adding up to six borders in all.

In the classroom or studio setting, the transitional names are left to the students to choose depending to how they experience those qualities kinesthetically and with different degrees of exertion or speed of transition. Pedagogically, these are major physical and cognitive learning experiences. The students themselves “own” the emotions generated by the physical experiences. This is the source of H’Doubler’s conception of art-making in movement.

## Patterns of Rhythmic Movement

Over a period of 40 years’ teaching dance, and choreographing some 90 works for professional and college ensembles, Wilson developed many exercises, including the one described in the preceding paragraph, based on the premise that, *in the art of the dance*,^8^ *rhythm is experientially elastic and is fixed to a meter only in closed pattern form.*

It is generally assumed that dance is anchored to the meters by which the passage of time is marked in music; steps match meters. A waltz is anchored to 3/4 time. A march is anchored to a duple meter—2/2 or 2/4 time. A jig’s meter can be marked in 2/4 or 6/8 time; but no matter how it may be counted, a jig is a jig is a jig.

But it is in the movement qualities that dancers make any beat elastic. Waiting in the wings of a stage, the dancer prepares to enter with an uplifting hop (in ballet called *temps levé)* and appears on stage already running, leaping, skipping, or hopping. The dancer’s qualitative exertion—elegantly performed—extends her or his off-stage experience into an entire phrase of movement that may or may not step to the music’s meter. Experience is elastic by virtue of its quality—sustained, pendulous, abrupt, maybe even vibratory, but most likely transitional between primary qualities.

Formed or choreographed works, whether of the fine art or exhibition variety, are rhythmically organized in one of four general structures. In larger works, different general structures are created and presented as ‘movements’ within a piece.

*a.* Open rhythm is common to sports, games, acting, and miming. Dance improvisation is by nature open rhythmed. Each dancer, as a soloist or as a member of an ensemble, moves freely in her or his personal rhythm. This is the ideal of expressing one’s “natural rhythms” advocated by Colby and H’Doubler. In concert dances such rhythms can be incorporated in the choreography and either “set” or remain spontaneous for performance.

*b.* Closed rhythm is set from start to finish. Closed rhythm is standard for classical ballets, for dance hall and ballroom dancing, for movie dance numbers (think “Singin’ in the Rain”), and for such popular dancing as swing, jitterbug, the twist, etc. Accompanying music or sound match the movements. Most Western meters are two, three, four, six, or eight beats to the measure. Slavic and East European folk dances are often in five, seven, nine, or more beats to the measure; Flamenco and Kathak in 12-beat measures, etc. Tempos might vary within measures; but the rhythms maintain their temporal characteristics.

*c.* Cumulative rhythm is an aggregate progression of disparate rhythms—often personal. They move toward an űber rhythm that no single rhythm can fully express. Stage plays are examples of this. Each character brings her or his personal rhythm to the story. Psychological tensions rise toward a climax as its many rhythms come toward each other in a complex crescendo. Think of the final scene of “Hamlet” or “Some Like it Hot.” It makes no difference whether a play is a tragedy, a comedy, a history, a melodrama, or a farce. The over-all pattern of cumulative rhythms is the same though the stories and characters are different. Each character is unique to begin with, but as the action progresses the characters interact and begin to create a larger rhythm in which they play a part cumulatively.

As for concert modern dance, choreographers, following the lead of Merce Cunningham, sometimes use aleatoric, better known as “chance,” methods for their works. From mid to late twentieth century and still in our day this method is increasingly used to achieve highly abstract works in a new aesthetic appropriate for our time. What appears to be chaotic or random is part of a larger order that peaks in an expanded consciousness.

Choreographer George Balanchine broke with ballet’s classical tradition with his use of cumulative rhythm in his modern works like “Agon” and “Apollo.” The steps and body positions performed by the dancers are clearly from the classical tradition—except for some idiosyncratic “Balanchinesque” embellishments like hyperextended wrists and heel bumps on the floor. The dancers keep to their closed rhythms while the music keeps to its closed rhythm—but they are not the *same* rhythm.

*d.* Phrased rhythm is most often used for concert modern dance, but also in music composed in the 20th and 21st centuries. American composer Charles Ives makes extensive use of phrased rhythms in his Symphony #4. Many modern choreographers deliberately do not “dance to the music”—which is an old aesthetic known as “music visualization” (considered *passé* and mildly degrading to *the Art of the Dance*). The dancers move in rhythmic phrases—but not metered phrases like Balanchine’s—simultaneously or coincidentally with the music or in silence. Rhythm and tempo might not be considered at all during certain phrases of the dance. How does one dance with the music of, say, Anton Webern or Karlheinz Stockhausen? It is the dancer’s movement qualities that create the dance, sometimes with, sometimes without meter. Sometimes the dance and music phrases cross or crisscross by choreographic design or by chance. Those times are like nodes in a temporal tapestry, a “partnership of temperaments” as Dalcroze instructor, John Colman, has said^9^. Such nodes are not necessarily metric. Frequently they are emotional.

The use of movement qualities can be seen and, certainly, heard in music. One should watch a string quarter or quintet by Schubert or Brahms and witness the vibrations of the left hand fingers, the sustained drawing of the bow sounding a harmonic, or watch the rapid changes of motion at the elbow. Often the entire torso sways in pendulous oscillations. The primary movement qualities provide the base for the emotional expressions of the transitional qualities.

All the performing arts employ the movement qualities to achieve emotional expression.

## Measured Energy as Phenomenon

As a student of Wilson’s, Henley recognizes in the description above an approach to rhythm that he experienced as a student but did not fully comprehend at the time. Wilson’s class would often begin with creative modern improvisation in, as Wilson names it, an “open rhythmic” structure. The purpose was to attune, first to the awareness of our own energy, then begin to measure that energy in conversation with the musician and fellow dancers. Students would learn complex multi-metered “closed rhythms” that were impossible to count. If counted, you were already behind. You had to feel the rhythm, you had to be the rhythm. In one exercise, dancers would slowly walk across the floor, moving the arms in a single arc from the sides of the body, to the front of the body at sternum height, then opening to the side and floating back down to the starting place. This task is deceptively simple as the movement itself is not complicated, but attention to phrasing one’s energy across the full space of the studio is quite challenging. Each of these experiences offered students an opportunity to attend to how we were, as H’Doubler would put it, measuring our energy.

Henley also has vivid memories of learning movement material and then having a fellow student, ask for clarification of the counts; to know which movements happen on which counts. Often, Wilson would offer a response similar to, “Let’s do it a couple times and we will find it together.” This was frustrating because part of the definition of being a good dancer, for Henley and his peers, involved doing the right move at the right time. This qualification for being a good dancer implied that a good teacher should, in turn, know the counts. These moments stand out because they were disequilibrating. Wilson either did not know or did not want to tell the dancers the counts for the movement phrase. He was urging the students away from superimposing counts on the movement and to discover and experience the energetic topography of the dance before tying it to an externally derived temporal structure. This is not to say that counting is bad, and certainly students did a lot of it in his class, but by removing the counts we were able to explore the rhythm of the dance as measured energy, or exertion, rather than than as alignment with meter.

Henley’s experience as a student rippled into his professional career as a teacher, where he perceived that many students believed that dance was something to be accomplished from the outside, rather than experienced from the inside; something to be watched and judged rather than experienced and explored. Henley’s practices in Wilson’s classes deeply informed how he approached this dynamic in his own classes, as he incorporated improvisation as a form of awareness building and opportunity to “find the counts” in some of the phrase work throughout the class.

He also began to draw on Maxine Sheets-Johnstone’s *Phenomenology of Dance* [2015 (1966, 1979, 1980)] as she made explicit experiences that had been implicit in his own work and his work with Wilson^10^. In her seminal dance philosophy text, Sheets-Johnstone argues that the phenomenological experience of dancing is of the creation of a dynamic line. Rather than dancing through space and time, the dancer creates a spatiality and temporality that has an inherent qualitative dynamic. In the experience of performance, the dance does not exist separately from the dancer. The dance and dancer are a form-in-the-making, an ongoing spatial and temporal revelation, and what the dancer creates and sustains is an illusion of force. In this definition, Sheets-Johnstone adopts Langer’s (1953) position that, in art, we encounter not the real world, but an illusion or semblance of the real world. In dance, in particular, the illusion is of the symbolic form of human feeling as revealed through force in movement. If we translate Sheet’s Johnstone’s force to H’Doubler’s energy or Wilson’s movement qualities, then we begin to see the congruence. The phenomenological experience of dancing is not one of making shapes through space and time, but of creating a spatial unity and temporal continuity through energy realized as a dynamic line.

Within this broader definition, Sheets-Johnstone (2015) argues that rhythm is an inseparable component of the dynamic line, an “interplay of forces which rise and fall, recoil and expand, which have sudden shifts in direction, which are now vigorous, now flaccid, and so on” (p. 84). Further, “each successive movement creates a qualitative change, and this, in turn, creates an accentual pattern, changing intensities, within the dynamic line” (p. 85). Reading this description of rhythm evoked, in Henley, kinesthetic memories of experiencing Wilson’s primary and transitional movement qualities. This rhythmic nature of the dynamic line is experienced pre-reflectively, or intuitively. It is part of the unity of the form-in-the-making. To count the meter, measure durations, or mark accents are reflective practices. They account for the temporal structure of movement, but “the dance does not come alive until the dancer passes beyond a mastery of the structure, and comes to realize the dynamic flow inherent in the total piece” (p. 88).

Sheets-Johnstone’s argument made explicit the trends Henley described earlier for dancers to want to layer rhythm onto the movement from the outside, reflectively, by aligning movements with counts, rather than experiencing it pre-reflectively, as already integrated into and inseparable from the dancing. To be clear, we all have experience of measuring our energy in patterned ways, and certainly, students in all dance classes have had experience creating a dynamic line in which rhythm was not measured but experienced. If they danced salsa with their mother around the kitchen, if they joined a hip-hop cypher, if they went to the dance club with their friends, they know what it is like to experience rhythm pre-reflectively. There is an aura, though, in the formal modern/postmodern academic dance classroom, both in pedagogical culture and aesthetics, that leads some students to believe that they need to reflectively know the counts rather than experience the dynamic line in order to “get it right.” In Supplementary Appendix, Henley offers Experiences 1–3 which facilitate students’ prereflective awareness of measured energy, the reader is encouraged to try these as a solo or group practice.

## Measured Energy as Process

The description above, inspired by a phenomenological approach to dance, prioritizes the individual’s lived experience of rhythm. Henley has also become interested in approaches to rhythm that decenter the individual and consider how the experience of rhythm is influenced by or even constructed by sociomaterial contexts. For instance, if a dancer is asked to improvise and thereby generate an energetic topography, we might wonder how the construction of the physical space prefigures the energetic choices of the individual, architectural rhythms. We might also wonder about what types of movements have historically been done by this group, cultural rhythms, and how gender or age might affect expectations for how energy is measured, ontological rhythms.

In each of these scenarios, the intentions of the individual are reprioritized and considered within a situated and dynamic sociomaterial context. This approach aligns with process philosophy, an intellectual tradition that “opposes ‘substance metaphysics”’ or descriptions of reality as “collections of static individuals” (Seibt, 2017). Rather it seeks to define reality as dynamic, changing, and in the process of becoming. In her text *Relationscapes*, Erin Manning (2012) explores the nature of rhythm through reference to the sculpture of Umberto Boccioni. She describes that he does not sculpt the body moving, but rather movement itself, which happens to include a body. The body is described as a collection of potentials. Process is metaphysically prioritized over matter. In this construction, force or energy prefigures action, the environment is alive with potential and in the experience of dancing, the dancer and the sociomaterial environment combine in a machinic interface to produce the event of the dance.

Manning uses Boccioni’s phrase “pure plastic rhythm” (15) to suggest that we exist in a world of malleable energy. Rhythm is an inflection of potential between body and environment such that the space both affects the dancer and the dancer affects the space. The dancer creates the space and the space creates the dancer. Before the dancer even enters the room there is already a rhythm (e.g., patterns of light on the wall, the wood grain of the floor, the clicking noise of the heater, and the flows of conversation between other dancers). No matter what the dancer’s individual intentions are in generating a rhythm they are in a sociomaterial context in which patterns of energy are structured and being structured around them. In Supplementary Appendix, Henley offers Experience 4, which facilitates the reprioritization of the phenomenological self in the emergence of bodily rhythms.

In these phenomenological and process philosophy informed approaches to rhythm, Henley aims to disrupt students’ prioritization of “knowing the counts” as a reflective activity and offer them the opportunity to experience temporality as flows of energy. Though these experiences are offered as improvisational exercises, it is possible to then transfer this awareness to set choreography, in which, yes, the dancer must “do the right move at the right time.” With this new awareness, however, that accomplishment is a side effect of attending to and following the energetic flow, or dynamic line of the dance.

## Conclusion

In this article, the authors have put forth a definition that originated with Margaret H’Doubler, that rhythm is measured energy. Wilson expanded on the relationship between measured energy and kinesthesis through the study of muscular activity and observation of dancers dancing. He identified four primary movement qualities that, when performed, evoke empathetic response in the performer and viewer. In this way, measured energy both scientifically and experientially describes rhythm. Henley extended Wilson’s approach by describing how the principle of measured energy was further developed through Sheets-Johnstone’s phenomenological description of rhythm as flows of force experienced through pre-reflective temporality, and Manning’s description of the ways in which we construct, and are constructed by, rhythms already embedded in the material and social world.

We developed the premise that action is not exclusively a response to rhythmic stimulus, but can itself be a rhythmic output which is developed through kinesthetic awareness to be used for aesthetic purposes. The periodicity or relative duration of movement qualities, perceived through kinesthetic awareness, are a schema around which a dancer can perceive and enact the energetic topography of a dance, or, put another way, the dance’s rhythm. Though we have put forth approaches to rhythm here, rooted in more deeply understanding qualities of movement and particular perceptions of the passage of time, we recognize that across time and geography, patterns will shift and change. Fundamental to our argument is that this phenomenon is experiential. We therefore invited the reader to engage in the movement exercises provided in Supplementary Appendix. We hope, that in the study of rhythm, researchers will seek a bridge between the scientific and the experiential.

## Author Contributions

Both authors listed have made a substantial, direct, and intellectual contribution to the work, and approved it for publication.

## Conflict of Interest

The authors declare that the research was conducted in the absence of any commercial or financial relationships that could be construed as a potential conflict of interest.

## Publisher’s Note

All claims expressed in this article are solely those of the authors and do not necessarily represent those of their affiliated organizations, or those of the publisher, the editors and the reviewers. Any product that may be evaluated in this article, or claim that may be made by its manufacturer, is not guaranteed or endorsed by the publisher.

## Footnotes

1 Margaret Newell H’Doubler (1889–1982) was born to Charles and Sarah H’Doubler in Beloit, Kansas. Margaret was a tomboy and the third of four children. They were raised in Warren, Illinois where the family moved for better financial prospects. Four years later they moved to Madison to be near the University of Wisconsin. Margaret entered the university in 1906 where she studied biology and philosophy. She was active in women’s sports—particularly basketball in which she excelled. In 1910 she graduated with a B.A. with honors in biology. She was hired as a teaching assistant in the Department of Women’s Physical Education to teach women’s sports and to coach the women’s varsity basketball team. Her position was elevated to instructor in 1911.

2 Visiting the private studios, Maragaret found that most classes were rudimentary ballet that she did not approve of. They did not, to her mind, promote the students’ awakening to their inner person. Ballet was too rigid. There was no play and no free exploration of movement. Occasionally she attended a modern dance (also known as “free dance” or “contemporary dance”) concert in the city or classes given by the rising professionals of the time. These performers represented the “new dancing” that Miss Trilling referred to. Again, Margaret was not pleased. She saw only unabashed egos and their imitators on display—no values for college women to cultivate.

3 But that program, just 3 years old, was evaluated by the overseers and found not to meet the criteria set for the Speyer School (originally a Settlement House built in 1906 for children) where the program was carried out (Spiesman, 1960). Those criteria were: “To design and develop a [dance] program that would be natural and free for [all] children; to permit self-expression; to harmonize (…) with the total school program.” The program was ended in 1916. However, Colby was hired by the Teachers College to continue experimenting with and developing a new progressive dance program not in the style of commercial studios.

4 From H’Doubler interview with Wilson (1966, Book 1, p. 23).

5 Though improvisation was a new element in the discipline of dance training, and it revealed to the experienced dance teacher’s eye what the individual students’ personal rhythms might be, no one had figured out how to “rhythmatize” them—to make them *rhythmically unified*. Even progressive teachers resorted to known folk dances and their rhythms which might not capture or stem from the student’s personal rhythms. This surmise was made by the graduate students in Miss HuhDee’s seminars.

6 American philosopher and scholar, John Dewey (1859–1952), was an influential faculty member at Columbia and a frequent lecturer at Teachers College. Margaret, who had been exposed to progressive education thinking at UW-M, and by Trilling in particular, was drawn to Dewey’s theories on democracy and experiential education. Her own thinking and values were growing, becoming loftier, during her two difficult years in New York. Eventually, her standard lead-in to her classes and speeches became: “In all of time there is but one of you. To find the quickening spirit that is you is your life’s work”.

7 H’Doubler interview with Wilson (1966, Book 1, p. 22).

8 The first known use of the definite article with an art form was in *The Iliad* (Homer, 1865, ca. 950 B.C.E., Bk I, l. 729). It remains the only art form so honored. H’Doubler never knew where the reference to “the dance” had come from - it is a common aesthetic expression; but she always saluted “the dance!” when a libation was raised at a picnic or high social gathering.

9 Colman interview with Wilson (1966, Book 1, pp. 53-54).

10 Sheets-Johnstone studied with H’Doubler in the 1950s and 1960s and received her Ph.D. from UW-Madision with a major in philosophy and a minor in dance.
