# Supplementary material for: Experiencing Rhythm in Dance
Source: Front Psychol. 2022 May 30;13:866805. doi: 10.3389/fpsyg.2022.866805 (PMC9195508; doi:10.3389/fpsyg.2022.866805)
Supplement: Supplementary file 1 [file Data_Sheet_1.docx]

Wilson Henley Experience Rhythm Supplementary Material

# Appendix

Principles that the dance teacher could keep in mind while designing exercises and experiences for the students in their technique, composition, or rhythm and movement classes:

a. Begin each class (yes, ballet too) with a timed improvisation: two to three minutes with light accompaniment for beginners, three to four minutes with sparse accompaniment for intermediate students, five or more minutes mostly in silence and wordless singing for advanced students. Be sure to begin the improvisation yourself, then observe.

b. Provide for kinesthetic learning. Reduce demonstration by the teacher or models. Full demonstration once or twice at the beginning of a new exercise or combination should be enough except in beginners’ classes. Thereafter, corrections like what foot to be on or the arm movements help the students find their way through the whole exercise kinesthetically.

c. Devote parts of some classes to tactility in conjunction with rhythmic accompaniment. Clear rhythmicality is closely tied to precise tactility. A good exercise is to stamp a foot and clap the hands at precisely the same instant. The effect of this is to commit every living molecule of the mind and body to the experience of the beat. This can also be done as the students do triplets across the floor: whole foot down on beat one, half toe on beats two and three and repeat the length of the floor. A clap on each beat ties the whole body together in sensing the beat.

Most people think that rhythm is wholly aural so they wait to hear the beat, even for just a part of a second, and follow it with the clap or step. This drags the tempo down. As the students get better at the exercise, the clapping can be dropped and free arm action can be added. Then accompaniment can be dropped out. In the near silence, except for the whisper of feet touching the floor, an established tempo can be heard and maintained. If students are still listening for a beat to move them along, the tempo will drag. A favorite instruction to the students as they dash across the floor is “Don’t follow the beat. BE the beat!”

d. Do not allow the students to rely constantly on mirrors for their appearance during barre and standing exercises. As the dominant sense order, vision stifles tactile and kinesthetic information. Mirrors are best used for correcting posture, arm and foot placement; but each correction should be followed up with eyes-closed kinesthetic and tactile replication of the corrections. “Now move your arms to second position. Now to third. Now fifth,” etc. This is a way to help build body image — not just in imagination but in fact.

e. Warm down each class with light improvisation ending in uplifted arms, upturned face Isadora style. Join in, then thank the accompanist with hand clapping.

On these principles and with experience and patience, most dance teachers can build their exercises and movement combinations quite well for the population they are serving — and feel joy daily!

## Using the Autosphere

Begin by exploring each of the primary movement qualities separately. This could be done with whole body movement or, if it is more comfortable, with one or both hands.

Sustained: continuous movement with no acceleration or deceleration.

Pendulous: oscillating movement continuously accelerating and decelerating.

Abrupt: movement continuously starting and stopping.

Vibratory: continuous rapid quaking as antagonistic muscles contend against each other.

For each one, explore increasing and decreasing your perceived sense of exertion.

Explore the transitory qualities by beginning with one then transitioning to another, for instance sustained to pendulous. Explore each combination and, as you do increase and decrease your perceived sense of exertion.

## Experience #1: Pre-reflective Temporality - Reflective Temporality

Frame: There are times we experience the passage of time subjectively - seconds feel like hours, hours feel like seconds - we are calling this pre-reflective temporality. There are times when we objectively measure time - watch a stopwatch, count along to a musical meter - we are calling this reflective temporality.

Part 1- Pre-reflective: Play several of your favorite songs with a distinctive beat or memorable melody. As you listen, you are invited to try to “be” the music. It doesn’t matter what movement you do; it could be swaying, stepping, clapping or movement vocabulary from a specific dance genre. Don’t worry about what you look like or if you are “on the beat” simply try to move as if you were the music. Afterward reflect on your experience of time and how you organized or patterned your energy. (In these experiences the reflective prompt is: What did you notice? Connections are then sought between the response and the frame.)

Part 2 - Reflective: Locate or create a complex patterned sequence of accents within a set meter (this is what students would likely call a rhythm, but that language is not used here as this article argues rhythm is more than this). In the past, “The Augurs of Spring” from *The Rite of Spring* by Stravinsky (credit to Paul Moore for this idea), has been useful, but most folk dances have complex and fun accent patterns as well. Create a visual depiction of the pattern with the meter numbered and the accents marked. (e.g. **1** ee **&** ah 2 **ee** & **ah**, where the bolded syllables are accented). First learn the pattern through counting and clapping, then explore the pattern through movement of your own choosing. Afterward, reflect on your experience of time and how you organized your energy.

Comparison: Reflect on the different modalities of pre-reflective and reflective temporality. How do they feel similar or different? When and where in your life do they normally occur? What are the relative strengths of each modality and when might they be usefully deployed for different purposes?

## Experience #2: ABA - Open/Closed/Open – Closed/Open/Closed

Frame: Although musical patterns can direct our energy, as they did in the previous exploration, we are also able to create our own patterns through how we organize our energy.

Part 1: Select or create music that has either an open or closed rhythmic structure. Open rhythm, according to Wilson’s definition above, is free of a consistent pulse. Closed rhythm has relatively stable repetitive patterns. Organize a playlist so that it follows this structure: A: song with open rhythm, B: song with closed rhythm, A: song with open rhythm. When the playlist is started, try not to imitate or embody the music, but to imagine that you are another instrument playing on top of the music. Afterward, reflect on your experience of time and how you organized your energy.

Part 2: Is the same as Experience 1, except the soundscapes are inverted so that the experience moves from closed to open and back to closed. Afterward, reflect on your experience of time and how you organized your energy.

Comparison: Reflect on the two experiences. How did you organize your energy between the two? Which type of soundscape (open/closed) did you prefer? Why? What responsibility do we have as dancers to not just follow along with the music, but to be creators of rhythm?

## Experience # 3: Open Movement Rhythm/Closed Musical Rhythm - Closed Movement Rhythm/Open Musical Rhythm

Frame: In the previous experience, you related your experience of measuring energy to music, that had both open and closed structures. We could also organize our energy in counterpoint to the music.

Part 1: Select or create a song that is distinctly closed rhythm. As the music plays, move with open rhythm against the closed rhythm of the music. Afterward, reflect on your experience of time and how you organized your energy.

Part 2: Select or create a song that is distinctly open rhythm. As the music plays, move with closed rhythm against the open rhythm of the music. Afterward, reflect on your experience of time and how you organized your energy.

Comparison: Reflect on the two experiences. How did you organize your energy? Which juxtaposition (open/closed) did you prefer? Why? How might these juxtapositions serve a choreographic purpose?

## Experience #4: Room Read (extended)

Frame: When generating movement, dancers often think that the dance must spring internally from our imagination. In Doug Varone’s Choreographic Device, Room Read (Varone, 2020), this tendency is disrupted as we begin to attend to the rich inspiration for movement embedded in the space around us.

Part 1: Create a series of 8 movements in response to 8 aspects of the room you are in. For instance, seeing a clock could result in a large circling of the left arm imitating the pathway of the clock hand, or it could result in small pulses of the shoulders for each second passing. Seeing a green backpack could result in miming putting a pretend backpack on or it could result in full body undulations as the dancer embodies their version of “green-ness”. Recreating shapes is a good place to start by try to also attend to textures, colors, temporalities, functions, and other characteristics of the environment that might inspire movement. It is also helpful to accumulate the movements, perhaps creating 1-3, then rehearsing those before moving on to 4-6 etc. Afterward, reflect on your experience of relating to the room and how you organized your energy.

Part 2: Experience 1 can be challenging as the tendency for many of us is to judge the movements that we make as good or bad. In Room Read, part of the task is to let the space tell you what to do next, and remove the internal judgment. In subsequent iterations, the time between deciding can be sped up and/or the need to remember the choices can be removed. The task becomes one of interfacing with the environment in an ongoing process of becoming.

Part 3: Once you are comfortable with the process, the space in which it is done can be altered. You can work in spaces that are large or small, indoor or outdoor, densely or sparsely decorated, familiar or unfamiliar. After each iteration, reflect on your experience of relating to the room and how you organized your energy.

Part 4: Instead of the material environment, bring your attention to the social environment. Attend to interacting with others in an ongoing process of becoming. Instead of physical qualities such as shape, color, and texture, respond to relational qualities. Afterward, reflect on your experience of relating to the room, the people in the room, and how you organized your energy.

# References

Varone, D. (2020). *Home.* Doug Varone and Dancers. Retrieved 15 December 2021, from Dovadance.org
